# Unifying Large- and Small-Scale Theories of Coordination

**DOI:** 10.3390/e23050537

**Published:** 2021-04-27

**Authors:** J. A. Scott Kelso

**Affiliations:** 1 Human Brain & Behavior Laboratory (HBBL), Center for Complex Systems and Brain Sciences, Florida Atlantic University, Boca Raton, FL 33432, USA; jkelso@fau.edu; 2 Intelligent Systems Research Centre, Magee Campus, Ulster University, Derry~Londonderry BT48 7JL, UK

**Keywords:** biological coordination, Haken–Kelso–Bunz, Kuramoto, complex systems, coordination dynamics

## Abstract

Coordination is a ubiquitous feature of all living things. It occurs by virtue of informational coupling among component parts and processes and can be quite specific (as when cells in the brain resonate to signals in the environment) or nonspecific (as when simple diffusion creates a source–sink dynamic for gene networks). Existing theoretical models of coordination—from bacteria to brains to social groups—typically focus on systems with very large numbers of elements (*N*→∞) or systems with only a few elements coupled together (typically *N* = 2). Though sharing a common inspiration in Nature’s propensity to generate dynamic patterns, both approaches have proceeded largely independent of each other. Ideally, one would like a theory that applies to phenomena observed on all scales. Recent experimental research by Mengsen Zhang and colleagues on intermediate-sized ensembles (in between the few and the many) proves to be the key to uniting large- and small-scale theories of coordination. Disorder–order transitions, multistability, order–order phase transitions, and especially metastability are shown to figure prominently on multiple levels of description, suggestive of a basic Coordination Dynamics that operates on all scales. This unified coordination dynamics turns out to be a marriage of two well-known models of large- and small-scale coordination: the former based on statistical mechanics (Kuramoto) and the latter based on the concepts of Synergetics and nonlinear dynamics (extended Haken–Kelso–Bunz or HKB). We show that models of the many and the few, previously quite unconnected, are thereby unified in a single formulation. The research has led to novel topological methods to handle the higher-dimensional dynamics of coordination in complex systems and has implications not only for understanding coordination but also for the design of (biorhythm inspired) computers.

## 1. Introduction: Biological Coordination


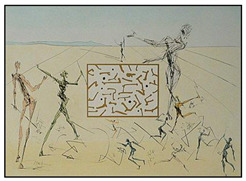


The engraving shown above is from a suite of 12 that Salvador Dali called “Hommage to Leonardo da Vinci”. This particular piece is called “L’Electronique”, the computer circuit. It evokes an abstract image of what the problem of biological coordination is all about. How can the dramatic postures and movements of the figures emerge from a box of so many very different and independently moving parts? The large male (?) figure to the right of the box of parts seems to be showing off. He seems to be saying “I know the answer!” However, to science, how coordinated movement emerges in natural systems containing so many degrees of freedom is still a bit of a mystery. Although we know more and more about the parts, at ever-increasing spatial and temporal resolution, it seems, their interdependence—the way they are coordinated—is not understood.

It is not that there has been a shortage of attempts to explain biological coordination by scientists and engineers. For example, the notion of *circuit* is popular in the field of neuroscience to account for orderly behaviors such as locomotion and even higher-order executive functions, such as decision-making. *Algorithmic* approaches from “good old-fashioned AI” [1] and its modern reincarnations are common in newer fields such as Cognitive Robotics. Engineers build circuits. Additionally, some neuroscientists engage in “circuit breaking.” Computer scientists build programs that tell circuits what to do. However, Nature, according to the theory developed here, fundamentally builds *synergies*—flexibly assembled patterns of coordination that are essential for a multiplicity of biological functions. Synergies offer a solution not only to the problem of coordination in living things but also to the origins of coordination. They are Nature’s way of handling biological and behavioral complexity. Without synergies and the forms of coordination they produce, life in all its variety would not, and could not exist [2,3,4].

How, then, does Nature build synergies? Where do they come from? The present article offers a perspective on these questions, and more particularly an explicit physical-based theory of how, on a very basic level, living things coordinate themselves. For the most part, the emphasis is on complex, but primitive forms of coordination relatively free of influences or impositions that might direct coordination for a specific function or purpose. Elsewhere we have referred to such spontaneously arising coordination tendencies as “intrinsic dynamics” and sought to understand how such intrinsic dynamics are modified, say, as an organism develops and learns or intentionally changes its behavior [5,6,7,8,9]. That is not the focus here, however. Rather, the aim is to elucidate the primitive nature of coordination unembellished by other, no doubt important influences upon which biological functions rely. The fundamental question addressed here is not so much one of “bringing together” or integrating more and more already organized functional parts. Rather it is how, in extremely complex systems, any set of constraints can synthesize out simpler forms of behavior. These simpler forms have complex, one might even say, universal dynamics. The ultimate goal, in short, aims at a physical foundation underlying Dali’s thought-provoking image.

## 2. The Birth of Coordination Dynamics: The HKB Model

Over 30 years ago, in a review article in the journal *Science*, Schöner and Kelso [10] reviewed the concepts, methods and tools of self-organization in open, nonequilibrium systems—at the time quite foreign to the behavioral and brain sciences —as a way to understand how dynamic patterns of coordination spontaneously form and change at both behavioral and neural levels of description. (Nowadays, the word self-organization is often used as a kind of vague, throwaway term that stands for spontaneous order and emergence in complex systems. The early work referred to here was aimed at establishing the concept of self-organization in biological coordination along with its predicted consequences.) Collectively termed *Synergetics* by Hermann Haken [11,12], one of the “fathers” of laser theory, who saw the crucial role of cooperative phenomena not just in physics but across the sciences [13], these ideas were used to show how a number of empirically observed phenomena in ordinary human movements (multiple stable states, transitions between them, hysteresis, etc.) can be mapped mathematically onto nonlinear dynamical laws. The latter in turn were derivable from lower levels of description. In an age where the dominant picture of movement’s control and coordination was thought to be by virtue of motor programs, schemas, reference-based cybernetic feedback systems and the like [14,15], this was a radical step that turned out to be a harbinger of paradigm change in the behavioral, brain, developmental and cognitive sciences [16,17,18,19].

It is a truism that reality can sometimes be found in the most ordinary phenomena. Galileo rolling a ball down an inclined plane comes to mind. Such was also the case for the original application of synergetic principles to biological coordination: namely, and somewhat incredibly, experiments on how humans coordinate the movements of their two hands [20,21,22,23,24]. The original motive behind these studies was that equine gait transitions, as when a pony spontaneously changes gait from a trot to a gallop, might be the biological equivalent of the phase transitions and cooperative phenomena seen in nonliving physical systems as when matter changes state or patterns form spontaneously in Nature [25]. The experimental window into this idea, ‘let your fingers do the walking,’ was to ask human subjects to rhythmically move their index fingers back and forth in parallel and then to ‘speed up’, i.e., make the movements of the two fingers faster. When starting the movements in a symmetrical pattern, both fingers flexing and extending at the same time, humans were able to perform across a range of movement frequencies from slow to fast. However, when starting in an asymmetrical pattern, one finger flexing at the same time as the other was extending, a dramatic effect occurred. At an apparently critical movement frequency, a spontaneous transition to symmetric finger movement was observed—highly evocative of gait transitions in quadrupeds and, for that matter, of nonequilibrium phase transitions in the rest of Nature [12,21]. The same phenomena were also observed at a neuromuscular level, obtained by recording from fine wire electrodes embedded (temporarily) in the relevant muscles [26]. An order parameter equation, which formed the top tier of the HKB model (after Haken, Kelso and Bunz [27]) and its stochastic equivalent [28] was shown to accommodate all the experimental findings—in addition to predicting new effects. In particular, the hallmark features of nonequilibrium phase transitions (themselves the core of self-organization in Nature) including a strong enhancement of fluctuations and critical slowing down—nowadays sometimes referred to as “anticipatory signatures” of upcoming transitions or “tipping points” [29,30]—were observed, quantified and modelled [28,31,32,33]. Notably, for the field of mathematical biology, which had struggled with these problems historically [34], the order parameter dynamics was shown to arise (meaning it could be analytically derived) from a nonlinear coupling of self-excited nonlinear (limit-cycle) oscillators. The latter were later shown to capture the flexion and extension movements of individual finger movements. In short, for biological coordination, concepts from physics such as order parameters and their essentially nonlinear dynamics were shown to rule at both collective and component levels. Thus was the Haken–Kelso–Bunz or HKB model born. Among other aspects, this work revealed limitations of the classical mechanical view that “Newton Rules Biology” [35]. Later research was to identify the neurophysiological basis of these phenomena in human brain recordings, both empirically (e.g., [36,37,38,39]) and theoretically [40,41,42,43,44,45,46].

Only recently (some 35 plus years after HKB) has it become clear that gait transitions in creatures such as mammals and birds are also nonequilibrium phase transitions and follow the laws of self-organizing coordination dynamics. Across nine species of mammals and birds, Granatosky and colleagues [47] showed that energy savings (metabolic costs of maintaining a gait) do not predict gait transitions, as was often thought (e.g., [48]). Rather their analysis demonstrated—like Kelso’s bimanual experiments and the HKB model—that animals change gaits to reduce or avoid unstable (highly variable) states of coordination. That is, when a given locomotor synergy is unable to meet current conditions and becomes too variable, creatures switch to another synergy that does meet the environmental challenge. Metabolic cost, rather than being the cause of gait transitions, is rather a reflection or a consequence of synergy (in)stability. Similar conclusions follow from the findings of Jantzen and colleagues [49] using fMRI BOLD imaging in humans to expose the neural regions involved in rhythmic, sensorimotor coordination. Creatures, by the way, are richly endowed with the capacity to sense proprioceptively and kinesthetically the synergic relations among the muscles, tendons and joints of single and coupled appendages (see [4] for an insightful treatment of the significance of kinesthesis). External information from sound or sight can also act to tune or modulate these synergies (e.g., [50,51]). When the sensed synergy is in danger of failing, and creatures cannot keep up with current demands, they switch gears. As the HKB model originally predicted, dynamic instability, a nonequilibrium phase transition in coordinated movement—and considered the most elementary form of self-organization in Nature [11,12,13]—is the mechanism that determines when animals decide to switch, causing transitions to happen. Unlike programmable machines, fluctuations are crucial to how this mechanism works (see, e.g., [52]). For basic forms of biological coordination, dynamic instability allows switching to occur without switches (cf. [53]).

## 3. The Extended HKB Model

Since its publication in 1985, there have been numerous empirical tests and theoretical extensions of the HKB model in a variety of very different systems and different levels of description from the neural to the social, attesting to HKB’s universality [54,55,56]. At the most elementary level, a key theoretical extension of the HKB model—again following experiments on sensorimotor coordination—was the incorporation of symmetry breaking [57,58]. This allowed for differences between the components (along with coupling strength) to be a determining factor in the governing dynamics [58,59,60]. The resulting interplay between the heterogeneity of the interacting parts and the coupling strength between them revealed novel but ubiquitous effects on the coordination dynamics, particularly metastability [2,6,61,62,63,64,65]. In the literature, this extended HKB model has come to be known as a basic law of coordination [2,66,67]. Deeper mathematical and empirical analysis has revealed a number of novel features and generalizations of the HKB model [68,69,70,71,72,73]. Though it is beyond the scope of this article to review all the evidence, the key point of extended HKB is that it is a law governing elementary forms of coordination that is independent of any specific material realization, attesting to its universality [6,67,74]. More important, perhaps, is that unlike the force-based couplings of classical mechanics, the coupling among biological components and processes in coordination dynamics was demonstrated to be informationally-based. That is, how things are coordinated depends on information exchange between the components mediated by sound, vision, touch, smell, kinesthesis and even emotion [75]. Again, informational coupling distinguishes coordination dynamics from the usual applications of classical mechanics. For example, when people say the great American gymnast Simone Biles “defies the laws of physics”, they are talking about classical mechanics. Simone’s fantastic tumbling is a coordinative feat governed also, one suspects, by the principles and mechanisms of informationally coupled self-organizing dynamical systems, i.e., coordination dynamics ([76]; see also [77]).

## 4. The Kuramoto Model

With few, but certainly notable exceptions—mostly concerning the classification of symmetries and their effects on the dynamics of animal and insect locomotory gaits ([78,79,80,81]; but see also [57,82,83]—much of the research supporting the extended HKB model has involved the coordination of two interacting components, whether these be two joints of a single limb, two limbs working together, coordinating a single limb with discrete auditory, visual and tactile stimuli, two persons interacting with each other, humans coordinating with a Virtual Partner controlled by the HKB equations, and so forth (see [2,56,67,84] for reviews).

In contrast, an entirely independent approach aimed at capturing statistical features of large-scale coordination among very many elements was developed by Kuramoto [85]. Kuramoto’s model was aimed first at explaining the onset of chemical oscillations, but in the hands of mathematical biologists like Steven Strogatz [86] and the late Art Winfree [87], it soon became a paradigm for large-scale coordination in complex biological systems that ranged from the flashing of fireflies, the firing of heart cells and neurons, to the awakened clapping of concert audiences composed of human beings ([86,88] for reviews). The fundamental idea of the Kuramoto model is that under certain conditions, a very large population of diverse elements undergoes a phase transition from incoherent, essentially random behavior to highly coherent behavior in which the entire ensemble coordinates as a synchronized unit—a basic synergy. Synchrony, from the subatomic to cosmic scales is considered one of the most pervasive phenomena in the Universe (News Feature Nature, 2003, 421, 780–782). In the context of understanding biological coordination, synchrony is well-known: though largely unacknowledged, it corresponds to what the great behavioral physiologist Eric von Holst [89] following his many studies of locomotion, called absolute coordination [6,90].

## 5. Coordinating the Few and the Many

**Figure 1:**
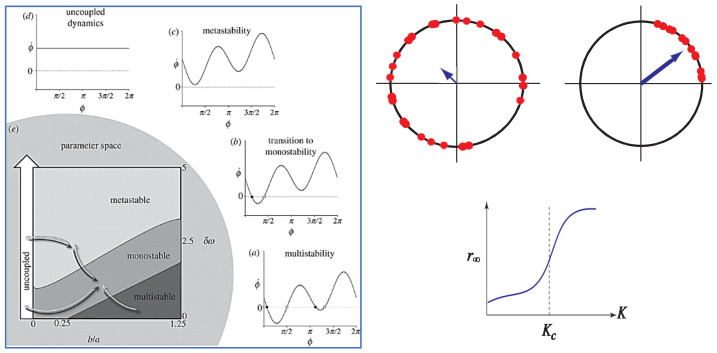
(**Left side**) shows the extended, symmetry breaking version of the HKB model of coordination for a large variety of dyadic systems. The time derivative of the HKB order parameter/collective variable relative phase plotted against itself displays the layout of the coordination dynamics. (a) and (b) illustrate the flow of the dynamics as the system switches from having multiple stable states (a) to just one (b). Mathematically, these transitions occur as the result of a collision between stable and unstable fixed points (filled and unfilled circles) that take the form of a saddle node bifurcation. In (c) there are no stable or unstable coordinated states at all, but just tendencies (or ghosts) of where the fixed points used to be. Such metastability occurs as a subtle balance of the coupling (b/a) between the components and the diversity or heterogeneity of the components (δω) in the parameter space shown in (e). (For completeness, (d) shows the flow of the uncoupled system). The parameter space (e) reveals a number of key features or “messages” of the essentially nonlinear coordination dynamics: (1) where the system resides in parameter space determines the kinds of coordination observed; (2) different paths in parameter space (shown by arrows) render a variety of coordination patterns possible, including transitions between them; (3) there does not need to be a direct one to one (input-output) relationship between parameter values and observed behavior. Shaded regions in (e) show that the same coordinated behavior may be observed over a broad range of parameter values, a condition known as degeneracy in biology [14,91]. Near boundaries, however, small changes in parameter values can have huge effects on the kind of coordination observed. (**Right side**) illustrates some of the key features of the Kuramoto model for an ensemble of very many rhythmic units or oscillators. Dots on the unit circles represent the oscillator phases. The size of the vector (shown by the length of the arrows) is the phase coherence, r which is often referred to as the Kuramoto order parameter, the multi-oscillator equivalent of the relative phase in the HKB model. The value of r is small when the oscillator phases are scattered, and large when the phases are clumped together and the system is synchronized. The plot of r against coupling strength K shows that when the coupling is weak the system is in a disordered (incoherent) state. When the coupling reaches a critical value K > Kc, the system becomes synchronized or absolutely coordinated. In statistical physics, this incoherent to coherent behavior is known as a phase transition.

The main features of the extended HKB and Kuramoto models are shown in Figure 1. 

It should be noted that the components in both the HKB and Kuramoto models are limit-cycle oscillators. Whereas in the case of HKB, much experimental work went into identifying the functional form of the oscillators and the nature of the coupling [59,92,93,94,95], this is not so in Kuramoto, where the limit-cycle nature of the oscillators is assumed and referred to, for obvious reasons, as a phase oscillator. As for the coupling, a good guess—originally by Winfree—is that each oscillator in the ensemble is affected equally by all the others. Thus, in the case of an audience that starts to clap in unison, each person is affected by the rhythmic applause of the whole room, not by the couple sitting next to them. Winfree was the first to realize that when the oscillators mutually synchronize, this is equivalent to a phase transition in physics. Just as water molecules freeze into ice in space, the oscillators line up in time—frozen, as it were, in a synchronized state. Note that in the Kuramoto model, the nature of the phase transition is always from disorder to order, whereas the nature of the phase transition in HKB is from one ordered coordination state to another (as in the anti-phase to in-phase transition in the original Kelso experiments) or from an ordered state to a partially ordered metastable form of coordination (as in later experiments of coordinating movements with visual and auditory stimuli). *Nota bene* (Figure 1c) there are no states at all in metastable coordination, only tendencies or dispositions due to the interplay between cooperative and competitive forces. Likewise, fluctuations do not receive a rigorous treatment in Kuramoto’s model, unlike HKB (e.g., [28]; see also [96]).

The Kuramoto and extended HKB models have a different ontological basis. The background to HKB, though biological, is based on the physics of living things [3,25,97,98]. Biological systems are never completely disordered or random in the sense of the mathematical modeling approaches of Kuramoto, Winfree and Strogatz. Rather, entirely in line with Haken’s physical approach and Synergetics [11,12,13], coordination dynamics views living things as fundamentally nonequilibrium systems, open to fluxes of energy, matter and information. Biophysically, for any process to persist, a cycle of work must be performed. According to Morowitz’s theorem [99] the flow of energy from a source to a sink leads to at least one cycle. Control and regulation are seen to occur by means of coupled limit-cycle oscillatory processes at all scales—Homeokinesis—in the terminology of Iberall, Yates, and colleagues [97,98,100]. Why, limit cycles one asks? Because limit cycles are needed to make up for dissipative losses that occur in all natural motions. Limit cycles are self-sustaining, autonomous, and operate independently of initial conditions. They exhibit temporal stability, and yes, mutual entrainment and synchronization. When nonlinearly coupled, they evolve new modes of coordination and effect transitions between modes [23]. In general, the synergies formed are softly assembled; coupling is by means of information exchange, not by forces. The emergence of new synergies or coordination patterns takes the form of nonequilibrium, synergetic phase transitions—entirely different from water turning to ice—accompanied by low-energy switchings into and among metastable modes. Acknowledging the danger of repeating oneself, the foregoing dynamical features of coordination have been observed experimentally again and again, at cognitive, behavioral, social and several neural levels (e.g., [2,55,56,64] for progressive reviews).

## 6. Toward Unification: The Marriage of HKB and Kuramoto

*“We dance round in a ring and suppose but the secret sits in the middle and knows.”*

(Robert Frost)

Though both extended HKB and Kuramoto models deal with basic coordination, it is not immediately obvious how they might be related to each other (but see [61,63]). One focuses on systems with very large numbers of elements (*N*→∞) and the other with just a few elements coupled together (typically *N* = 2). This raises an important question: How can the large-scale dynamics of the Kuramoto model (Equation (1)) be reconciled with the small-scale dynamics of extended HKB Equation (2)?

(1) $${\dot{\theta }}_{i}=\omega_{i}-\frac{K}{N}\overset{N}{\underset{j=1}{\sum }}\sin\phi_{ij}$$

where $\phi_{ij}=\theta_{1}-\theta_{2}$ and $\omega_{i}$ corresponds to the dispersion of natural frequencies, and

(2) $$\dot{\phi }=\delta \omega -a\sin\phi -2b\sin2\phi$$

where $\phi =\theta_{1}-\theta_{2}$, $\delta \omega =\omega_{1}-\omega_{2}$ is the difference in natural frequency between two oscillators, and *a*, *b* corresponds to coupling strength (see Figure 1).

It seems that we are faced with a dilemma. In the Kuramoto model (e.g., [96,101]), which deals with very many elements (in principle an infinite number), the possible microlevel coordination patterns are too numerous to be studied exhaustively, due to the high dimensionality of the phase space. Moreover, although there are many analyses of the Kuramoto model in the literature (see Discussion in [102]), none have included any empirical data on rhythmic coordination to justify their relevance to living systems. Experimentally, the large number of elements makes it difficult to perform systematic manipulations that scan the system’s repertoire of possible patterns. Low-dimensional (macro) measures such as the overall level of synchronization may adequately capture the system’s collective states (cf. Figure 1) but are insufficient to characterize coordinative complexity.

On the other hand, in systems with very few components typically treated by the extended HKB model, the repertoire of collective patterns and phase transitions may be fully explored with the help of experimental manipulations, but the system’s limited size may curtail the complexity of observable coordination. A saying of Otto Rössler’s comes to mind: experiment is an escape hole from the darkness of speculation. What is needed is an experiment it seems, not on a very large crowd (Equation (1)) and not strictly on dyads either (Equation (2)) but somewhere in between. To reconcile the many and the few, a way is needed to experimentally manipulate the system’s coordination dynamics on multiple spatial and temporal scales of description. Ideally, what is required is to study an ensemble of intermediate size that is big enough to reveal the system’s coordinative complexity, yet small enough to yield to experimental manipulation and analysis of its micro-dynamics.

To bridge the two-fold gap of system size and experimental accessibility and control, Mengsen Zhang and colleagues [103] studied rhythmic movement coordination in groups of eight human beings who were predisposed to move at the same or different frequencies. Both the Kuramoto and extended HKB models predict that the form and stability of coordination varies with the strength of coupling and the difference in natural frequency (so-called frequency predisposition) between components. On these grounds, Zhang et al. [103] hypothesized that manipulating the distribution of frequency predispositions (which they termed diversity) and coupling strength should produce different dynamic patterns of coordination and induce different forms of collective behavior. The frequency difference was chosen as a parameter to manipulate diversity within and between group members, not only because of its theoretical significance (Equations (1) and (2)), but also because it is possible to control systematically and measure quantitatively.

The actual experiment consisted of a so-called *continuation* paradigm (see [104] for the neural underpinnings of this paradigm) in which multiple human subjects (*N* = 8 per group) arranged in an octagon tapped together rhythmically on a set of touch pads [103]. This is called the continuation paradigm because each person in the group is first paced by a (here visual) metronome at a certain frequency for 10 s, after which the metronome is switched off and the task is to continue tapping for another 50 s. During the latter, any and all coordination within the group of 8 persons is by visual information exchange. (Of course, each person also receives tactile and proprioceptive information about his or her own movement, but this is not shared between individuals.) Although the subjects are physically isolated from each other, each person’s taps are seen by other members of the ensemble in real time as flashes of specific LEDs on an array in front of each subject (see [103] for details). This situation creates the possibility for subjects to spontaneously coordinate with each other, even though they are not explicitly instructed to do so. All a subject was told to do was simply maintain the original frequency of his/her own pacing metronome (hence the continuation paradigm). Diversity in the tapping frequencies was manipulated by pacing each subject for 10 s after which the metronome was turned off and they saw each other’s behavior for 50 s. Each subject was referred to by a number from one to eight, associated with a specific touch pad and LED assignment. The pacing frequency was always the same within the group of subjects numbered 1 to 4, and within the group of subjects numbered 5 to 8 but could be different between the two groups.

**Figure 2:**
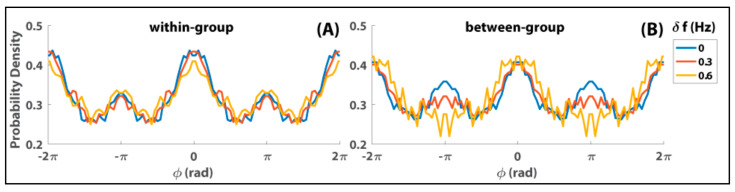
Relative phase distribution during episodes of strong interaction for within-group dyads (**A**) and between-group dyads (**B**) in the Zhang et al. experiment [103]. Notice the presence of both in-phase and anti-phase coordination within groups, and the progressive elimination of anti-phase between groups as a function of the frequency difference (δf).

There are many detailed results of this so-called “human firefly” experiment, but here I wish to draw on the ones that are central to reconciling models of the many and the few. Figure 2 shows the distributions of relative phase within episodes of strong interaction in the Zhang et al. [103] experiment. Such episodes correspond to 10,796 dwells in particular phase relations extracted from 258 trials (see Supplementary Information in [103]). The total time of these dwells corresponds to about 14% of the total time of interaction. Within groups (Figure 2A), both in-phase (peak near ϕ = 0) and anti-phase (smaller peak near ϕ = ±π) are dominant coordination patterns, with in- phase stronger than anti-phase, regardless of diversity conditions (δf in color). That is, regardless of whether subjects within a group were paced at the same or different frequencies, they tended to produce both in-phase and anti-phase coordination. Between groups (Figure 2B), the dominance of in-phase coordination was retained across different levels of diversity, but anti-phase lost its attraction with increasing diversity (gradually flattened peaks near ϕ = ±π from blue to red to yellow). Such a result is highly typical of studies of dyadic coordination (e.g., [105,106,107,108,109]) but here also applies to social interaction in mid-sized groups.

How might this result be explained? The Kuramoto model (Equation (1)) was developed to provide a mathematical interpretation of self-organized collective oscillation (Section 5.1 in [85]), guided by the “[en]slaving principle” discovered by Hermann Haken (Preface, Kuramoto [85]) Only the first Fourier mode was included in the coupling function for simplicity rather than for any empirical reasons (Equation (5.4.2) in [85]). Obviously, Equation (1) cannot handle the presence of both in-phase and anti-phase coordination and transitions between them as observed in the Zhang et al. [103,110] experiment.

Likewise, although two Fourier modes are present in Equation (2) to capture bistability and the bistable to monostable phase transition observed in human bimanual coordination, and although the symmetry breaking term is added to the original HKB equation to capture frequency differences between the components [58], Equation (2) can only handle dyadic (*N* = 2) situations.

The simplest model (Equation (3)) that captures all important observations in the mid-scale human experiment [103] on multiple levels of descriptions turns out to be a combination of Equations (1) and (2).

(3) $${\dot{\varphi }}_{i}=\omega_{i}-\overset{N}{\underset{j=1}{\sum }}a_{ij}\sin\left(\varphi_{i}-\varphi_{j}\right)-\overset{N}{\underset{j=1}{\sum }}b_{ij}\sin2\left(\varphi_{i}-\varphi_{j}\right)$$

Here, $\varphi_{i}$ is the phase of the *i*th oscillator, $\omega_{i}$ the natural frequency, $a_{ij}>0$ and $b_{ij}>0$ govern the coupling strength.

Obviously, Equation (3) becomes the classical Kuramoto model when the second-order coupling term *b* in Equation (3) is equal to zero. This would be fine if only in-phase coordination was observed experimentally, but this is not true. As shown in Figure 2, when it comes to the distribution of interpersonal relations there is a preference also for anti-phase which diminishes as a function of the diversity between components. Equation (3) therefore is a natural generalization to arbitrary N of the extended HKB equation (Equation (2)).

**Figure 3:**
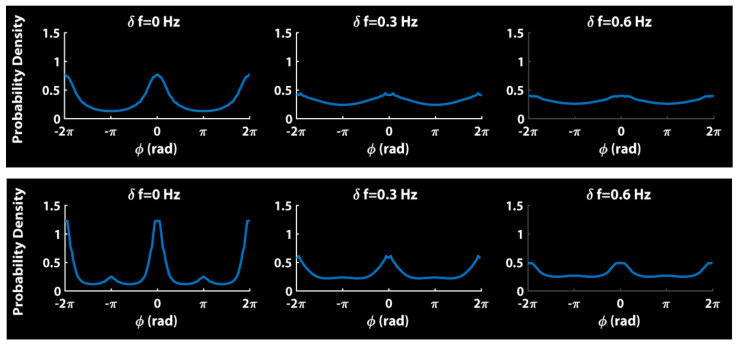
Relative phase distributions of simulations of Equation (3) aggregated over 200 trials with *b* = 0 (**top row**) and positive values of *a* and *b* (**bottom row**). The probability density of relative phase is plotted as a function of frequency diversity (δf = 0, 0.3, 0.6 Hz). Adapted from [102].

Relative phase distributions of simulations of Equation (3) bear this picture out [102]. Figure 3 shows the distributions for each diversity condition (δf = 0, 0.3 and 0.6 Hz). The top row is the classical Kuramoto model, with *b* = 0. Notice the absence of anti-phase.

The bottom row shows simulations of Equation (3) with second-order HKB coupling, i.e., positive values of *a* and *b*. Notice the presence of both (strong) in-phase and (weaker) anti-phase coordination which diminishes with increasing frequency diversity. The comparison of the qualitative features shown in Figure 3 to actual data (e.g., Figure 2) is impressive (see also, Figure 5 in [102]).

**Figure 4:**
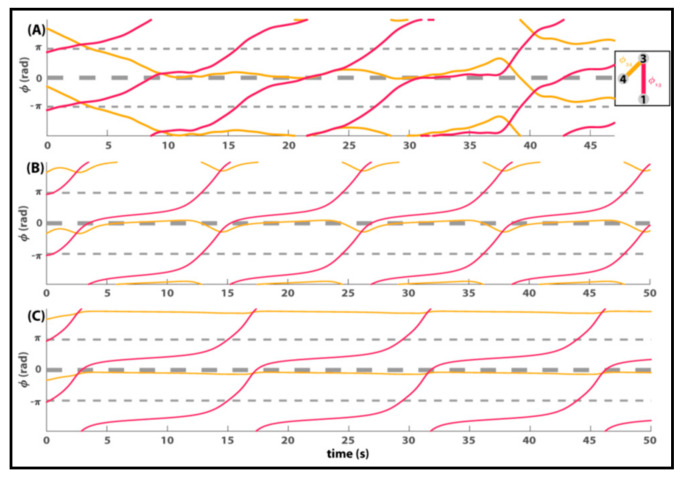
How coordinative relations (relative phase trajectories over time) are affected by nonuniform coupling between three interacting subjects (**A**) or (**B**,**C**) three agents according to computer simulations of Equation (3). The small box shows the experimental arrangement. In (**A**), subjects 1 and 3 are metastable and 3 and 4 are in-phase for most of the trial. See text for detailed discussion. In (**B**), agents 3 and 4 are nonuniformly coupled (*a3* > *a4*) with all other couplings smaller and equal. In (**C**), agents 3 and 4 have equal and greater coupling (*a3* = *a4*) than all others. The point is that Equation (3) produces the detailed microdynamics among interacting components. Adapted from [102].

Many of the phenomena in the original Zhang et al. experiment [103] are captured by uniform coupling. A beauty of Equation (3) is that it also handles the detailed coordination dynamics between components that are not uniformly coupled. An example is shown in Figure 4 (see also Figure 6 of Zhang et al. [102]). The top row shows actual data between 3 subjects spatially configured as illustrated in the small box. The relative phase between subjects 3 and 4 stays at in-phase most of the time before switching to anti-phase. Notice the relative phase is perturbed a bit (3 bumps around 10, 20 and 35 s) before switching. The relative phase between subjects 1 and 3, however, exhibits a dwell~escape behavior, characteristic of metastable coordination dynamics ([6,64,65]. Temporary dwells near in-phase are followed by phase wrapping escapes. It is quite clear that the two dyadic behaviors are related. When 1 and 3 dwell briefly near zero, 3 and 4 are perturbed. In fact, it looks as if the interaction between 1 and 3 induces critical slowing down in 3 and 4 [32,33,37]. The longer 1 and 3 dwell, the greater the effect on 3 and 4, until their relation switches and they go out of phase with each other. 3 and 4′s relationship, it seems, has clearly been affected by 1′s relationship with 3. This finding that the joining of a new member (e.g., person 1) induces changes in pre-existing coordinative relations (e.g., dyad 3–4), strongly suggests that multiagent coordination is more than the mere sum of isolated dyads.

The second and third rows of Figure 4 show two simulated trials of Equation (3) with identical initial conditions and natural frequencies, estimated from the human data. In Figure 4B, agent 3 is more “social” than agent 4 (*a3* > *a4*). More precisely, agent 3 has a much stronger coupling than all others. The recurring bumps between agents 3 and 4 are nicely reproduced. In Figure 4C, agents 3 and 4 are equally coupled and remain in-phase throughout the run.

## 7. The Generalized HKB Model of Coordination Dynamics

The Kuramoto model emphasizes incoherent to coherent synchronization as a phase transition, where the latter is in-phase ‘synch’ only (see Figure 1). There is little or no discussion of bi- or multistability, metastability, order–order transitions, etc. Indeed, the emphasis on synchrony is so strong, that anti-phase coordination is referred to as “anti-synchrony” [86]. Analytic work on the Kuramoto model is mostly done on an infinite number of globally coupled oscillators, incorporating some further mathematical assumptions (see [102] for review).

On the other hand, the extended HKB model works well for dyadic dynamics and has been demonstrated to be crucial to explain bi- or multistability, phase transitions, metastability, hysteresis, etc. observed in many kinds of coordination in different biological systems and on different levels. The work referred to here on mid-sized ensembles captures key features of human social coordination that transcend both Kuramoto and extended HKB models. In particular, it shows that the detailed aspects of dyadic coordination are present also along with other key aspects of biological coordination such as spatiotemporal metastability and multiple timescales of interaction (e.g., Figure 4). This middle ground experiment reveals the full complexity of biological coordination and is captured by Equation (3), a generalization of the extended HKB model for *N* > 2. The higher-order coupling of HKB is required for complex phase clustering (multistability) and switching (mostly through heteroclinic orbits—another mechanism for metastability, cf. [111]). A theme that emerges is that many, maybe all coordination phenomena arise from the interplay of just two factors: the coupling of the elements and the diversity between them. The former has the potential to create collectives and the latter to oppose them. This generalized HKB model may thus be viewed as a “coordination of coordinations” or a synergy of synergies [112]. For instance, as illustrated in Figure 4, the triadic relations within 8-person coordination constitute a particular coordination itself.

It is worth emphasizing that the basic experimental work that motivated the model is on human beings—who are far from idealized oscillators, except when they have to be. Although people can easily synchronize to an external stimulus, the picture is rather more complicated when the latter is removed and they have to move on their own, influenced only by their visually mediated interactions with others. The fact that the Generalized HKB model handles how a diverse group of complex (human) agents interact with each other when their actions influence—and are influenced by—the rest of the ensemble in unpredictable ways, is a key step toward embracing coordinative complexity.

## 8. Relevance of Generalized HKB to Small and Mid-Size Group Coordination

As stated up front, the present paper follows Pattee’s dictum [113] that “searches for the *origin* of coordination must be of a different type than searches for existing structures and functions” and that “we must try experiments with little or no implications of functional behavior” (p. 170). The experiments reported here probe basic forms of coordination somewhat independent of functional activities such as singing or marching or rowing a boat together. A curious person, however, might wonder how the Generalized HKB model of coordination dynamics relates to the myriad examples of group and collective phenomena observed in nature and studied experimentally in numerous fields and contexts. This is a burgeoning area of research that spans many areas of investigation and can only be touched upon it here. A classic, of course, is William H. McNeil’s (1995) “Keeping Together in Time” in which the profound role of shared rhythmic movement in creating and sustaining human communities throughout the ages is articulated in detail under the hypothesis that emotional connections—which can promote trust, affiliation and cooperation—are formed by “muscular bonding” (see [114] for a modern and informed analysis, and [115] for how collective music listening can promote synchrony). Such emotional connections between agents have been demonstrated to affect the stability of coordination, e.g., in the dyadic case of a human interacting with a virtual HKB model ([75]; see also [116] for review). The entire issue of bio-behavioral synchrony between on-line physiological measurements and behavioral processes (e.g., [117]; see also [118] for an example of the group case) is open to the present multiscale theoretical modeling approach. Moreover, viewed from the ubiquitous dwell~escape metastable coordination observed in our experiments and model, the episodic nature of bio-behavioral rhythms warrants close investigation beyond synchrony itself. The same of course can be said about the coordination of brain rhythms [65,119,120]. 

As Alderisio and colleagues [121] note: “Synergetic movements of two or more people mirroring each other frequently occur in many activities such as handling objects, manipulating a common workpiece, dancing, choir singing, and movement therapy” (e.g., [122]). Their approach extends the HKB framework to networks of HKB oscillators coupled via various functions. Although the emphasis thus far is on analytic conditions for synchronization, preliminary comparison with data from the Richardson et al. rocking chairs experiments ([107]; see also [123,124]) suggests that connecting the oscillators through nonlinear HKB coupling works best. Interestingly, nonlinear HKB coupling also works best in the Nalepka et al. [125] model of their shepherding experiments, perhaps attesting to the original proposal of Jirsa et al. [46] that the HKB coupling is quite fundamental in situations in which switching occurs from one coordinative state to another (though time delay coupling is likely to be important as well [40,126]). One can obviously delve into the deeper layer of the HKB model, namely the rich space of nonlinearly coupled nonlinear oscillators. Again, this has been the subject of much research—for example by the Amsterdam group of Beek and colleagues (e.g., [92,93,127])—and could be a topic for a complete review on its own (e.g., [128]).

The present paper shows how the extended HKB and Kuramoto models can be reconciled. For reasons already discussed (Section 2, Section 3 and Section 4) the key move was to go from HKB towards Kuramoto. However, if one ignores the details of the oscillators, the opposite direction is possible too. For example, Dotov et al. [129] reduced the Kuramoto model to two coupled phase oscillators in their studies of mutual synchronization between auditory stimuli and human gait. This allowed them to explore how the bidirectional coupling between walking and an auditory beat induces spontaneous entrainment to a faster cadence as well as overcome initial disparities between preferred gait (step cycle) and stimulus frequencies. Their model has a single stable fixed point corresponding to a synchronized state, the locus of which depends on the relative coupling strengths between stimulus and participants. The most effective auditory-motor synchronization occurs when both stimuli and step cycle adapt to a common tempo. The present Equation (3) allows for nonuniform coupling of this kind (cf. Figure 4) as well as other possibilities when one goes beyond the case of two phase oscillators and first-order coupling.

Recent work of Bardy et al. [130] on mid-size groups bears a strong relation to the present theoretical framework, and coordination dynamics in general (see also [131]). Bardy et al. are concerned with how groups of 7 people achieve the goal of unison/synchrony despite perturbation and parameter changes such is altering the spatial configuration of the group. Moving in unison, as Bardy et al. [130] note, is either the goal or clearly contributes to it, and results from both (i) personalized characteristics and (ii) the way individuals are coupled together. Their valuable review of the numerous variables that influence (i) and (ii) could constitute a major research program in its own right. We note that (i) and (ii) are precisely the determining factors in the extended version of HKB (Equation (2)) for both intrapersonal and interpersonal coordination, and of course are a key aspect of the merger of extended HKB and Kuramoto (Equation (3). Alderisio et al. [131] show that a network of coupled heterogeneous oscillators (a version of Kuramoto) accommodates their experiments on group synchrony. As shown in Equations (2) and (3) for small and mid-size group coordination respectively, with HKB coupling rather more than unison/synchrony is possible, both theoretically and experimentally. For example, the predicted multi- and metastability (indicative of intermittent forms of coordination) as well as transitions are obvious features to explore in these kinds of settings.

Last but not least (and by no means all) are remarkable works on collective motion in human crowds (e.g., [132,133]) and the vibrant field of research on animal collectives (e.g., [134,135]). There too, global patterns of coordination are seen to emerge from local interactions through the process of self-organization. Warren and colleagues’ approach, like ours is experiment-driven, with the aim of building pedestrian models (typically in couples) to explain emergent crowd behavior from the bottom-up. At least abstractly, and likely not by chance, one can see similarities between their approach and that adopted here—namely from nonlinearly coupled dynamical systems (HKB) to statistical mechanics (Kuramoto). However, as here, details matter, e.g., the definition of the neighborhood of interaction, the nature of the coupling between individuals and so forth. These remain to be worked out.

Couzin’s and others work on the self-organization of coordinated spatiotemporal patterns in group-living vertebrates—from fish schooling to bird flocking and migrating herds and beyond—is extraordinary (e.g., [135] for review). With respect to the present theory, Couzin [134] notes how synchronization may enhance the efficiency and/or the collective computational capabilities of animal groups. Honeybees, for example, exhibit a remarkable collective response in response to the threat of predatory wasps. According to Couzin, “patterns of synchronous activity have been found in almost every animal group studied, from the simplest multicellular animals (Placozoa) to humans. Synchrony plays a role (over a wide range of timescales) in almost every aspect of group behavior” (p. 844; see also [136] pp. 162–165). Although an emphasis on synchrony seems justified, the ignoring of “anti-synchrony” is not. There may well be other generic properties of the Generalized HKB model (Equation (3)) that remain to be discovered, including multistability, chimeras, waves, phase transitions and metastable coordination patterns. Recent research [137] shows, for example, that even in a group of 8 *identical* (nano) oscillators (unlike the humans in our work), exotic dynamical states can occur (as in the patterns of coordination observed and modeled in our work). Hamlet’s words “there are more things in heaven and earth, Horatio…” resonate strongly. They are an inspiration to probe further.

## 9. Some Implications and Future Directions…

The unification of Kuramoto and extended HKB models sheds some insight into certain fundamental issues raised in Schrödinger’s famous book “What is life?” and the commentaries 50 years later (see, e.g., [3]). Therein, Schrödinger asserted that the disorder–order mechanism of statistical mechanics was inadequate to explain life and that a ‘new’ order–order mechanism was required (see also [138]). The Kuramoto model provides a disorder–order mechanism, i.e., for the onset of synchrony as a phase transition. Adding the second-order coupling of HKB, provides the order–order mechanism identified in Kelso’s phase transition experiments and in the later work of Zhang et al. [103] on intermediate-sized ensembles. Thus, the marriage of Kuramoto and extended HKB (Equation (3)) provides both a disorder–order and an order–order mechanism that covers all the observed effects as well as predicting other, quite salient features of biological coordination—particularly the metastable mode that sits in between partially ordered and fully ordered states of coordination, reflecting both cooperative (integrative) and competitive (segregative) processes.

The Kuramoto model is in frequent use in current neurobiological modeling (e.g., [96]) and analysis of EEG and fMRI brain imaging (e.g., [139,140]). In particular, the Kuramoto order parameter or synchronization index—an average taken over all oscillating elements—is frequently used as a measure of functional connectivity among brain regions. The empirical and theoretical work reviewed briefly here suggests that not only synchronization, but multistability and metastability are ubiquitous features of coordination that operate on multiple timescales and are likely to be highly relevant for understanding the brain [6,62,64,65,120]. Rhythms permeate the brain: the millisecond temporal resolution of EEG expresses the rapidly changing electrical dynamics of neuronal populations. Although, as Buzsaki and Freeman [141] note, not everyone agrees that brain rhythms are critically important, everyone acknowledges that neuronal activity should be coordinated across neurons and structures. The fact that brain rhythms are conserved across species [142] makes them highly likely candidate entities for the multiscale, synergistic theory of coordination dynamics proposed here.

Even in the ‘human firefly’ experiments described briefly in the present paper, the complexity of behavior among an ensemble of 8 interacting agents is already high. It is difficult to keep track of all the interactions even in this relatively simple case. Recent work suggests that the path of the ensemble is dictated by the ensemble’s coordination topology rather than its inter-agent interactions. Thus, the very many different ways that agents interact or relate to each other may be underpinned by a common topology [143]. Such multiscale topological portraits highlight collective aspects of coordination patterns that are irreducible to properties of individual parts. This makes the challenge of identifying collective variables in the complex data and systems encountered in science and engineering all the more important (for an excellent recent review of the concept and the challenges it presents in the field of skilled movements, see [144].) Topological methods may provide new insights into a system’s coordination dynamics. At the very least, an integration of the tools of applied topology and the concepts of coordination dynamics seems warranted. 

The unification of Kuramoto and extended HKB and the range of phenomena accounted for hints strongly of overarching principles underlying coordination in living things. Is it always the case that new features emerge as one moves from one scale to another? [145]. The present theoretical model incorporates coordination phenomena observed at small, intermediate and large scales. The basic elements of the theory are nonlinear, limit-cycle oscillators. Whether these elements are molecules, macromolecules, cells, brain regions, body parts, organisms, stimuli and responses does not matter, so long as the basic description is in terms of a phase or limit-cycle oscillator. This has been a basic assertion of a thermodynamically based, intrinsically nonlinear, physical biology and has been at the heart of our approach to coordination since the get go. The original HKB model, for example, proposed an explicit functional form for both the (nonlinear) oscillators and their (nonlinear) coupling. 

So, how might the very basic, physical picture of coordination presented here connect to the teasing image of Dali shown at the beginning? It seems like a long stretch from a box of many moving parts to the dynamic posings and accentuated movements that are expressed in Dali’s art. An inroad might be to study in detail the underlying dynamics of such stylistic balletic movements. The late Armin Fuchs and the author did this with quite remarkable results [146]. We invited the author’s God daughter, Makaila Wallace, then recently retired as chief ballerina in the BC Ballet (Canada)—and previous to that the Royal Swedish Ballet—to choose a dance of about 20 s duration and to perform it under a number of different conditions, e.g., with and without music, fast versus slow, expressing different emotions, sad, happy, fearful, etc. We recorded her movements with a VICON motion capture system composed of 8 infrared cameras and 33 infrared markers attached to her body. Each marker provided positional information of x, y, z coordinates and was sampled at 100 Hz, thereby allowing reconstruction of her movement trajectories in three-dimensional space over time. The covariance matrix constructed for the dancer’s arms and legs is symmetric with real and non-negative eigenvalues and orthogonal eigenvectors that represent basic or prototypical movement patterns (eigenvalues being measures of how much a given pattern contributes to the variance in the original, high 99-dimensional time series). Principal Component Analysis revealed—for both the dancer’s arms and legs—that the top eigenvalues were for coordination patterns that were either in-phase or anti-phase. Complex coordination was classified as one or the other along three different directions in 3D space. It seems that in-phase and anti-phase dominate even very sophisticated forms of biological coordination.

How does our basic theory handle such extremely complex and high-dimensional coordination characteristic of ballet? The answer appears to lie in Nature’s synergies, themselves constituted of very basic symmetries (in-phase and anti-phase), whose dynamics are low-dimensional. Both the data and its analysis support the hypothesis that such synergies are the building blocks of coordinated behavior at all scales. 

Throughout the present paper we have stressed the basic coordination problem, namely how coordination comes about, its origins, and how to understand it when we see it. Pattee [113] addressed this problem but offered no solution. What Pattee did realize was that the problem of coordination’s origins is not solved by calling upon natural selection. Some minimal level of coordination is needed in the first place for natural selection—and other processes such as development and learning—to act upon. And they do. Traces of their beginnings are felt at the highest level of artistic expression.

## Data Availability

Not applicable.
